# Misfolded alpha-synuclein detection by RT-QuIC in dementia with lewy bodies: a systematic review and meta-analysis

**DOI:** 10.3389/fmolb.2023.1193458

**Published:** 2023-05-17

**Authors:** Carmen Peña-Bautista, Rakesh Kumar, Miguel Baquero, Jan Johansson, Consuelo Cháfer-Pericás, Axel Abelein, Daniel Ferreira

**Affiliations:** ^1^ Alzheimer’s Disease Research Group, Health Research Institute La Fe, Avda de Fernando Abril Martorell, Valencia, Spain; ^2^ Department of Biosciences and Nutrition, Karolinska Institutet, Stockholm, Sweden; ^3^ Division of Clinical Geriatrics, Center for Alzheimer Research, Department of Neurobiology, Care Sciences and Society, Karolinska Institutet, Stockholm, Sweden; ^4^ Neurology Unit, University and Polytechnic Hospital La Fe, Valencia, Spain

**Keywords:** dementia with Lewy bodies, alpha-synuclein, RT-QuIC, biomarker, diagnosis, copathologies, prodromal, REM sleep behavior disorder

## Abstract

**Introduction:** Dementia with Lewy Bodies (DLB) is the second most common cause of neurodegenerative dementia after Alzheimer’s disease (AD), but the field is still lacking a specific biomarker for its core pathology: alpha synuclein (α-syn). Realtime quaking induced conversion (RT-QuIC) has recently emerged as a strong biomarker candidate to detect misfolded α-syn in DLB. However, the variability in the parameters of the technique and the heterogeneity of DLB patients make the reproducibility of the results difficult. Here, we provide an overview of the state-of-the-art research of α-syn RT-QuIC in DLB focused on: (1) the capacity of α-syn RT-QuIC to discriminate DLB from controls, Parkinson’s disease (PD) and AD; (2) the capacity of α-syn RT-QuIC to identify prodromal stages of DLB; and (3) the influence of co-pathologies on α-syn RT-QuIC’s performance. We also assessed the influence of different factors, such as technical conditions (e.g., temperature, pH, shaking-rest cycles), sample type, and clinical diagnosis versus autopsy confirmation.

**Methods:** We conducted a systematic review following the PRISMA guidelines in August 2022, without any limits in publication dates. Search terms were combinations of “RT-QuIC” and “Lewy Bodies,” “DLB” or “LBD”.

**Results:** Our meta-analysis shows that α-syn RT-QuIC reaches very high diagnostic performance in discriminating DLB from both controls (pooled sensitivity and specificity of 0.94 and 0.96, respectively) and AD (pooled sensitivity and specificity of 0.95 and 0.88) and is promising for prodromal phases of DLB. However, the performance of α-syn RT-QuIC to discriminate DLB from PD is currently low due to low specificity (pooled sensitivity and specificity of 0.94 and 0.11). Our analysis showed that α-syn RT-QuIC’s performance is not substantially influenced by sample type or clinical diagnosis versus autopsy confirmation. Co-pathologies did not influence the performance of α-syn RT-QuIC, but the number of such studies is currently limited. We observed technical variability across published articles. However, we could not find a clear effect of technical variability on the reported results.

**Conclusion:** There is currently enough evidence to test misfolded α-syn by RT-QuIC for clinical use. We anticipate that harmonization of protocols across centres and advances in standardization will facilitate the clinical establishment of misfolded α-syn detection by RT-QuIC.

## 1 Introduction

Dementia with Lewy Bodies (DLB) is the second most common cause of neurodegenerative dementia (Erkkinen et al., 2018). However, the diagnosis of DLB is complex and is mainly based on the core clinical features of visual hallucinations, cognitive fluctuations, REM sleep behaviour disorder, and parkinsonism (McKeith et al., 2017). Alzheimer disease (AD) or Parkinson disease with dementia (PDD) are common dementias that share clinical symptoms with DLB, which makes the differential diagnosis of DLB difficult [(Gomperts, 2016; Erkkinen et al., 2018; Walker et al., 2019)]. α-syn-related pathology is the key hallmark of DLB and its location defines different pathological subtypes [olfactory bulb, amygdala predominant, brainstem, limbic and diffuse neocortical] (McKeith et al., 2017). In addition, patients with DLB often show co-pathologies like AD and cerebrovascular pathology, which can influence the course of DLB (Jellinger and Attems, 2008).

**FIGURE 1:**
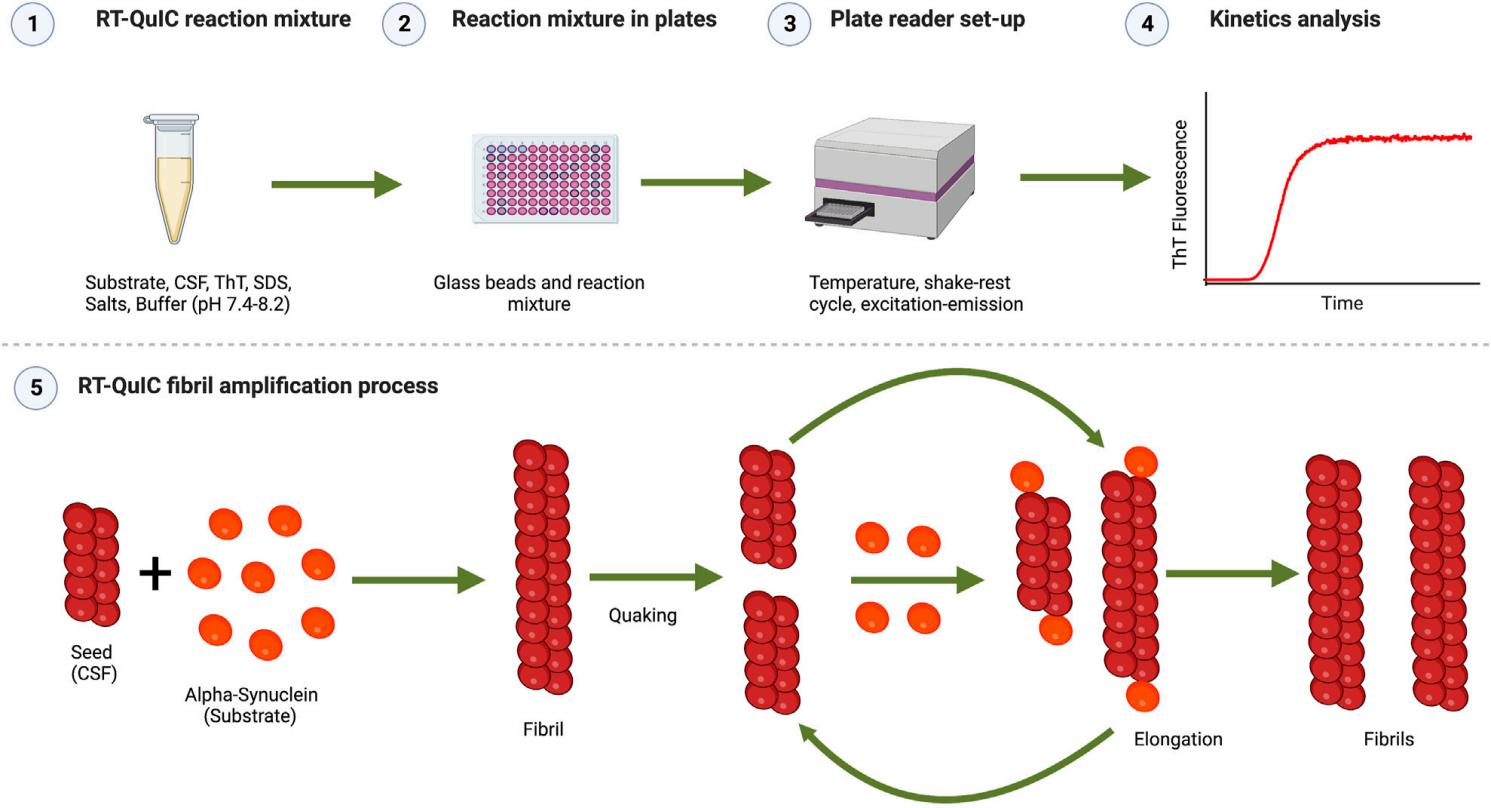
Schematic representation of the RT-QuIC assay. (1) RT-QuIC reaction mixture: Buffer at a selected pH (normally 7.4-8.2), Thioflavin T (for detecting fluorescence by binding to the aggregates formed during the reaction), additional additives (e.g., NaCl and SDS), recombinant α-syn monomers as the substrate, and CSF samples as seeds. (2) Reaction mixture in plates: the reaction mixture is pipetted in a 96-wells plate pre-loaded with glass or silica beads. (3) Plate reader set-up: the 96-well plate is inserted in a plate reader, where the assay conditions are fixed (temperature, shake-rest cycles times, excitation, and emission wavelength) and kinetic measurement is done. (4) Kinetics analysis: kinetic parameters (lag time, log phase, nucleation) analysis is performed on the kinetics data. The representation shows aggregation kinetics of α-syn showing the typical sigmoidal curve of Thioflavin T (ThT) versus time. ThT fluorescence curve has three main phases as lag phase (time which shows no increase in fluorescence), the exponential growth phase (the stage where new fibrils are rapidly formed which shows an exponential increase in fluorescence), and the stationary or plateau phase (occurs when the substrate has been consumed and there is no increase in fluorescence). (5) RT-QuIC fibril amplification process: α-syn seed (present in CSF) recruits α-syn monomer (substrate) and forms α-syn fibrils. Fibrils grow further during the rest phase by recruiting monomers. These fibrils break into a large number of small fragments following the quaking cycle acting as new seeds. These cycles are repeated during kinetics amplifying/accelerating the fibrilization process. Images were created with BioRender.com.

The current diagnostic criteria for DLB list several indicative and supportive biomarkers, including reduced dopamine transporter uptake in basal ganglia by SPECT or PET, abnormal 123 iodine-MIBG myocardial scintigraphy, polysomnographic confirmation of REM sleep without atonia, relative preservation of medial temporal lobe structures on CT or MRI, generalized low uptake on SPECT/PET perfusion/metabolism with reduced occipital activity, and prominent posterior slow-wave activity on EEG, along with recognition of an overrepresentation of GBA mutations in DLB (McKeith et al., 2017). However, all these are just indirect biomarkers that do not really assess the α-syn pathology directly. There is thus a lack of reliable direct *in-vivo* biomarkers of α-syn-related pathology. There has been an intense work in the field of a-syn PET tracers, while some challenges remain to be solved (Kotzbauer et al., 2017). Recently, the development and emergence of the so-called real-time quaking induced conversion (RT-QuIC) method has opened new opportunities in the field, showing a high potential for the diagnosis of α-synucleinopathies such as Parkinson’s disease (PD), PDD, multiple system atrophy (MSA), and DLB. RT-QuIC can detect the seeding activity of α-syn in different human samples (Orru et al., 2012; Fairfoul et al., 2016; Heras-Garvin and Stefanova, 2020; Nakagaki et al., 2021). RT-QuIC is based on the prion theory, which postulates that a pathogenic protein can induce the conversion of a “normal” protein into a pathogenic one, where the former acts as a seed that promotes conversion and aggregation (Soto, 2011). Applied to α-synucleinopathies, an abnormal alpha-synuclein conformer would induce or accelerate the conversion and aggregation of recombinant α-syn that acts as a substrate (see Figure 1 for a schematic representation). RT-QuIC is an evolution of the Protein Misfolding Cyclic Amplification (PMCA) assay, which includes changes such as replacing shake for sonication, reading by Thioflavin T (ThT) against Western blot, generating non-infectious products, and increasing speed without compromising sensitivity (Dong and Satoh, 2021) (Srivastava et al., 2022). PMCA and its variants were first developed for prion diseases and Creutzfeldt–Jakob disease detection, and then adapted for the detection of other substrates including PrP, α-syn, amyloid-β, and tau (Soto et al., 2002; Colby et al., 2007; Morozova et al., 2013; Salvadores et al., 2014). These adaptations thus allow the detection of different proteinopathies characteristic of neurodegenerative diseases, using the same samples.

α-Syn RT-QuIC has sparked great attention during the last years, leading to the publication of several reviews (Kurapova et al., 2022; Srivastava et al., 2022; Y; Wang et al., 2022). While these reviews provided important insights, they did not focus specifically on DLB, did not provide reports for different clinical stages nor stratify by the presence of co-pathology, and did not provide meta-analytical estimates of sensitivity and specificity of α-syn RT-QuIC. Hence, despite the promising results, the exact diagnostic potential of α-syn RT-QuIC in DLB is still unclear.

The current systematic review provides the state-of-the-art of α-syn RT-QuIC in DLB. Our two specific aims were: (i) to assess the diagnostic performance of α-syn RT-QuIC in DLB versus controls and other neurodegenerative groups, in prodromal stages of DLB, in the presence of co-pathologies, in different neuropathological DLB subtypes, and in DLB patients with different genetic variants—including meta-analytical estimates of sensitivity and specificity of α-syn RT-QuIC; and (ii) to investigate several factors known to influence the α-syn RT-QuIC’s diagnostic performance, by statistically comparing sensitivity and specificity values from studies including different clinical and technical conditions, sample types, and genetic factors.

## 2 Material and methods

### 2.1 Search methods

Following the PRISMA guidelines, we conducted a systematic review in PubMed, Web of Science (WoS), and Scopus in August 2022, without any limits on publication dates. The search strategy included the terms “RT-QuIC” and “Lewy Bodies,” “DLB” or “LBD,” with the following filters (“abstract” and “title” in WoS; “article” in Scopus). Additionally, relevant publications from articles’ reference lists were identified and included.

The inclusion criteria for the current review were studies that: (1) included α-syn seeding activity detection by RT-QuIC; (2) used a case-control design for either (2.1) a DLB group compared with control, AD, or PD groups; (2.2) DLB without versus with co-pathologies; or (2.3) included cases at prodromal stages of DLB (mild cognitive impairment of Lewy body type—MCI-LB—or iRBD) versus a control group; and (3) were published in English. Due to methodological variability across studies, the control group in 2.1. And 2.3. Could include neurological patients in some cases. We addressed this issue by annotating and reporting the inclusion of neurological patients in our review. Additionally, the PD group could include patients with PDD in some studies.

The exclusion criteria were studies that: (1) were reviews, abstracts to conferences, or case-report studies; (2) did not include the number of positive and negative α-syn RT-QuIC biomarker results for each study group; or (3) did not include DLB or prodromal DLB stages.

The selection of publications was carried out by a single researcher (CP-B) involving a second researcher (RK or DF) when needed.

### 2.2 Data collection, risk of bias and evaluation of methodological quality

A data extraction sheet was developed to collect relevant data including author and publication year, groups of participants and type of diagnosis (clinical/histopathological), sample type, co-pathologies, conditions of the technique (sample volume, number of beads, temperature, buffer, incubation, and reading conditions, salt and sodium dodecyl sulfate (SDS) concentration) and the proportion of positive and negative results for each group. Data extraction was carried out by a single researcher (CP-B) for each eligible study involving a second researcher (RK or DF) when needed.

**FIGURE 2:**
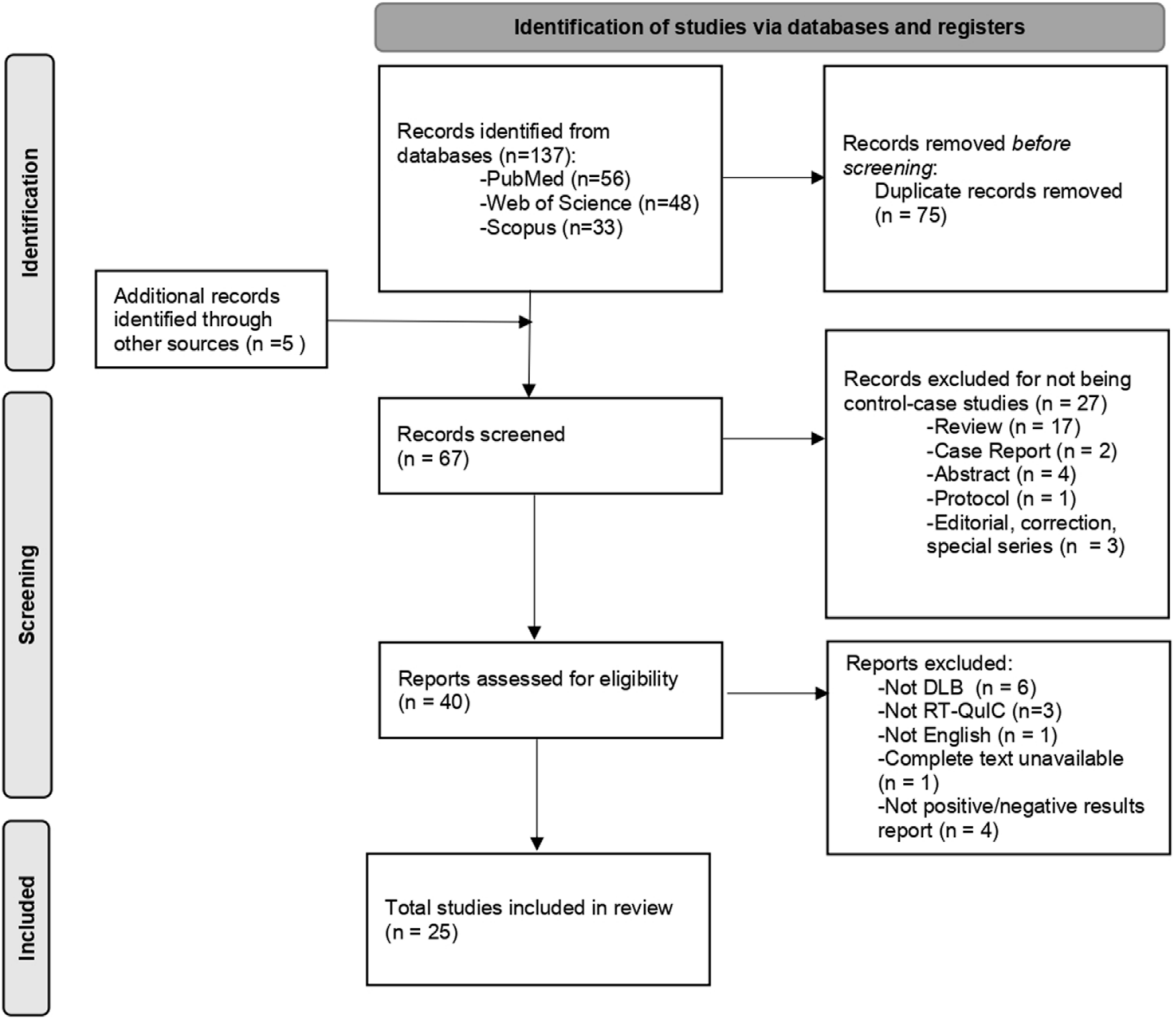
Flow diagram for the studies selection. DLB: Dementia with Lewy Bodies; RT-QuIC: Real-time quaking induced conversion.

Figure 2 shows the study selection flow. A total of 142 records were initially identified in the search. After removing duplicates, 67 articles were selected based on title and abstract, and 25 articles were finally included based on full-text screening. Of these, 11 articles were completely independent studies with no overlapping authors. For the other 14 studies, we could not assess the complete independence among the studies.

### 2.3 Outcome measures and statistical analysis

To address our first specific aim, for each comparison of diagnostic groups, we calculated the following diagnostic indexes: sensitivity, specificity, positive predictive value (PPV), negative predictive value (NPV), and proportion of correctly classified individuals. In addition, we carried out a meta-analysis for sensitivity and specificity values for the comparison between DLB and controls, DLB and AD, and DLB and PD, using the MetaDisc 1.4 software. We allowed the same study to contribute to meta-analytical estimates more than once if they provided data for different sample types, included cohorts with different types of diagnoses, or provided multiple data such as data collected in life and post-mortem or across different brain areas. When different groups were available, calculations were carried out on the most homogeneous groups, especially concerning the control group, where we favoured the selection of individuals without neurological or neurodegenerative diseases whenever it was possible. The meta-analysis was based on random effect models (DerSimonian-Laird), including estimations of 95% confidence intervals (CI) and generation of forest plots. We meta-analysed all available studies combined and afterwards provided separate estimates for CSF studies, due to their larger availability and clinical interest (as opposed to studies performed on biopsies).

To address our second specific aim for each comparison of diagnostic groups, we tested for statistical differences in sensitivity and specificity values using Mann-Whitney test when we had at least 5 studies in each separate condition. Otherwise, we tested the most common condition vs the rest of the conditions combined. Specifically, we performed comparisons for the type of diagnosis (clinical vs histopathological), sample type (CSF vs brain tissue vs skin vs olfactory mucosa), temperature (30°C vs 37°C vs 40°C vs 42°C), shaking-rest cycles (1 min shaking-1 min rest vs 1 min shaking-14 min rest vs 1 min shaking-29 min rest vs 40 s shaking-140 s rest), shaking speed (200 rpm vs 400 rpm vs 432 rpm vs 500 rpm vs 600 rpm), buffer concentration (40 mM vs 50 mM vs 100 mM vs 32% 5X Phosphate buffer), buffer pH (6.5 vs 7.5 vs 8 vs 8.2 vs 8.4), NaCl concentration (160 Mm vs 170 Mm vs 500 mM vs no NaCl), and SDS concentration (0.00125% vs 0.0015% vs no SDS). Statistical analyses for these comparisons were performed using IBM^®^ SPSS^®^ Statistics version 23 (SPSS, Inc., Chicago, IL, United States). *p*-values <0.05 were considered statistically significant in all the analyses.

## 3 Results

### 3.1 Systematic review—Summary of selected articles

Among the 25 included articles, 17 studies included a control group, 11 included an AD comparison group, and 14 included a PD comparison group. In addition, 6 studies included DLB groups without versus with co-pathologies, and 7 included data for prodromal DLB stages.

The most common sample types were CSF (*n* = 16) and brain tissue homogenates (*n* = 10). In addition, some studies included submandibular gland tissue biopsy (SMG) (*n* = 1), skin (*n* = 2), or olfactory mucosa (OM) (*n* = 2). Please see Table 1 for a description of the key methodological characteristics of the selected studies.

**TABLE 1:** Description of the studies in the systematic review, including methodological conditions.

Author, year	Neuropathological confirmation	Sample type	Sample volume (µL)	Beads	a-syn	Buffer	Incubation and reading	Salt or SDS
Rossi et al. (2020)	Partially^a^	CSF	15	6 silica (0.8 mm)	RH wt α-syn	40 mM PB (pH 8.0)	42 °C, 1′shaking-1′ rest (DO 400 rpm)	170 mM NaCl, 0.0015% SDS
Bargar et al. (2021)	Yes	CSF and Brain	2	6 silica (0.8 mm)	RH wt α-syn^b^	40 mM NaPO4 (pH 8.0)	42 °C, 1′shaking-1′ rest (DO 400 rpm)	170 mM NaCl, SDS 0.0005%
Groveman et al. (2018)	No	CSF	15	6 glass/silica (1 mm or 0.8 mm)	K23Q or RH wt α-syn	40 mM PB (pH 8.0)	42 °C, 1′shaking-1′ rest (DO 400 rpm)	170 mM NaCl, 0.0015% SDS (for CSF)
	Yes	Brain	2					
Mammana et al. (2021)	Partially^a^	CSF	15	6 silica (0.8mm/1 mm)	K23Q	40 mM PB (pH 8.0)	42 °C, 1′shaking-1′ rest (DO 400 rpm)	170 mM NaCl, 0.0015% SDS (for CSF)
		Skin	2					
Han et al. (2020)	No	Brain	2	4 glass (1.0–1.25 mm)	RH wt α-syn, A53T^b^	100 mM PB (pH 8.2)	37 °C, 1′shaking- 1′rest (DO 400 rpm)	Not specified
Fairfoul et al. (2016)	Yes	CSF	15	37 ± 3 mg silica (0.5 mm)	RH wt α-syn (Stratech)^b^	100 Mm PB (pH 8.2)	42 °C, 1′shaking-1′ rest (DO 400 rpm)	0.0015% SDS
Wang et al. (2021)	No	Skin	2	Not specified	RH wt α-syn (rPeptide/in-house)^b^	40 mM PB (pH 8.0)	42 °C, 1′shaking-1′ rest (DO 400 rpm)	170 mmol NaCl, 0.00125% SDS
Quadalti et al. (2021)	No	CSF	15	6 silica (0.8 mm)	RH wt α-syn	40 mM PB (pH 8.0)	42 °C, 1′shaking-1′ rest (DO 400 rpm)	170 mM NaCl, 0.0015% SDS
Hall et al. (2022)	Yes	CSF	15	6 glass (0.8 mm)	K23Q	40 mM NaPO4 (pH 8.0)	42 °C, 1′shaking-1′ rest (DO 400 rpm)	170 mM NaCl, 0.0015% SDS
Sano et al. (2017)	Yes	Brain	5	Not specified	RH wt α-syn	50 mM HEPES (pH 7.5)	40 °C, 40″shaking-140″rest (circularshaking 432 rpm)	Not specified
Iranzo et al. (2021)	No	CSF	15	37 ± 3 mg zirconium/silica (0.5 mm)	RH wt α-syn^b^	100 mM (pH 8.2)	30 °C, 1′shaking-14′rest (DO 200 rpm)	Not specified
Rossi et al. (2021)	Partially^a^	CSF	2	6 silica (0.8 mm)	RH wt α-syn	40 mM PB (pH 8.0)	42 °C, 1′shaking-1′ rest (DO 400 rpm)	170 mM NaCl, 0.0015% SDS
Bongianni et al. (2019)	Partially	CSF	15	6 glass/silica (1 mm/0.8 mm)	RH wt α-syn	40 mM PB (pH 8.0)	30 °C, 1′shaking-14′rest (DO 200 rpm)	170 mM NaCl, 0.0015% SDS
	Yes	Brain						
Manne et al. (2019)	No	CSF	15	6 silica (0.8 mm)	RH wt α-syn	40 mM PB (pH 8.0)	42 °C, 1′shaking-1′ rest (DO 400 rpm)	170 mM NaCl, 0.0015% SDS
	Yes	Brain	2					
Poggiolini et al. (2021)	No	Brain	15	Not specified	RH wt α-syn, truncated recombinant a-syn 1-130 and 1-115	100 mM PIPES (pH 6.9)	37°C, 1′shaking-15′rest (DO 500 rpm)	Not specified
Brockmann et al. (2021)	No	CSF	15	6 silica (0.8 mm)	RH wt α-syn	40 mM PB (pH 8.0)	42 °C, 1′shaking-1′ rest (DO 400 rpm)	170 mM NaCl, 0.0015% SDS
Candelise et al. (2019)	Yes	Brain	3	Not specified	RH wt α-syn^b^	32% of 5× PBS (pH 8.4)	37 °C, 1′shaking-29′rest (DO 600 rpm)	170 mM of NaCl
Sakurai et al. (2022)	No	CSF	15	37 ± 3 mg zirconium/silica (0.5 mm)	RH wt α-syn^b^	100 mM PB (pH 7.4)	30 °C, 1′shaking-14′rest (DO 200 rpm)	Not specified
Manne et al. (2020)	Yes	SMG	5	6 silica (0.8-mm)	RH wt α-syn	40 mM PB (pH 8.0)	42 °C, 1′shaking-1′ rest (DO 400 rpm)	170 mM NaCl, 0.00125% SDS
Jin et al. (2022)	Yes	Brain	5	6 silica (0.8-mm)	RH wt α-syn^b^	40 mM PB (pH 8.0)	40 °C, 1′shaking-1′ rest (DO 400 rpm)	170 mM NaCL, 0.0006% SDS
Shin et al. (2022)	Yes	Brain	2	4 glass (1.0–1.25 mm)	RH wt α-syn^b^	100 mM PB (pH 8.2)	37 °C, 1′shaking-1′ rest (DO 400 rpm)	160 mM NaCl, 0.00125% SDS
Shahnawaz et al. (2017)	No	CSF	40	Not specified	RH wt α-syn	100 mM PIPES (pH 6.5)	37 °C, 1′shaking-29′rest (DO 500 rpm)	500 mM NaCl
Stefani et al. (2021)	No	OM	2	37 ± 3 mg glass (0.5 mm)	RH wt α-syn	100 mM PB (pH 8.2)	30 °C, 1′shaking-14′rest (DO 200 rpm)	Not specified
Poggiolini et al. (2022)	No	CSF	15	Not specified	RH wt α-syn	0.1 M PIPES (pH 7.0)	40°C, 1′shaking-1′ rest (DO 500 rpm)	Not specified
Perra et al. (2021)	No	OM	2	37 ± 3 mg glass (0.5 mm)	RH wt α-syn	100 mM PB (pH 8.2)	30°C, 1′shaking-14′rest (DO 200 rpm)	0.0075% SDS (for CSF)
		CSF	15					

^b^ Commercial.

^a^ Partially: A subgroup of participants has neuropathological confirmation.

OM: olfactory mucosa; SMG: submandibular gland; RH: recombinant human; wt: wildtype, PIPES: piperazine-N, N′-bis(2-ethanesulfonic acid); DO, double orbital; SDS: sodium dodecyl sulfate.

### 3.2 Diagnostic capacity of α-syn RT-QuIC for DLB

#### 3.2.1 DLB versus controls

**FIGURE 3:**
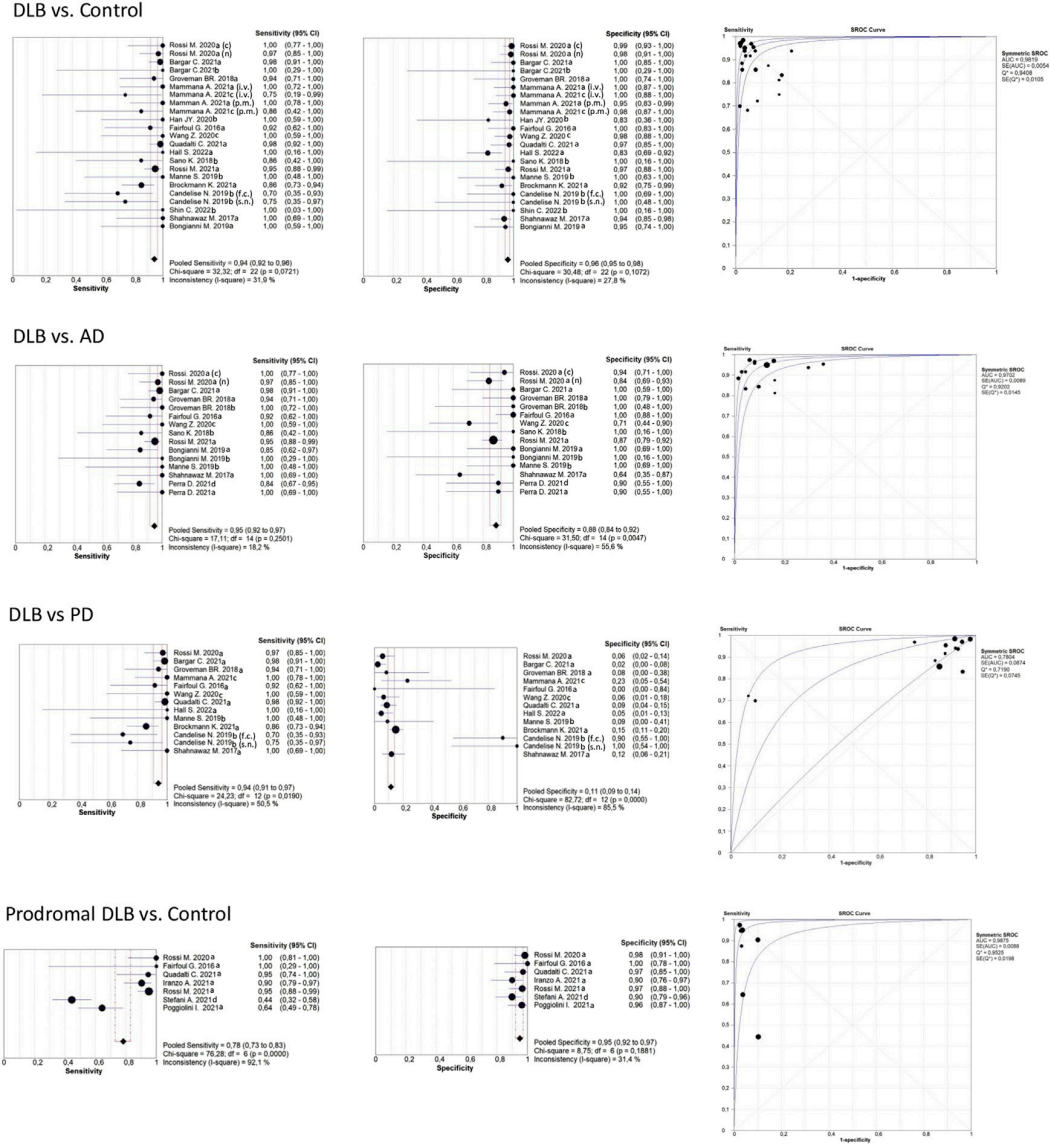
Forest plots meta-analysis of all sample types (CSF, brain, skin, OM). The references that appear duplicated provided independent data for sample type, type of diagnosis, or other conditions. These references are differentiated as follows: CSF; brain; skin; olfactory mucosa; f. c.: frontal cortex; s. n: Substantia nigra; i. v. *In vitam*; p.m: post-mortem (n) not neuropathologically confirmed; neuropathologically confirmed. For more detailed information see Tables 2, 3, 4, and 6.

**FIGURE 4:**
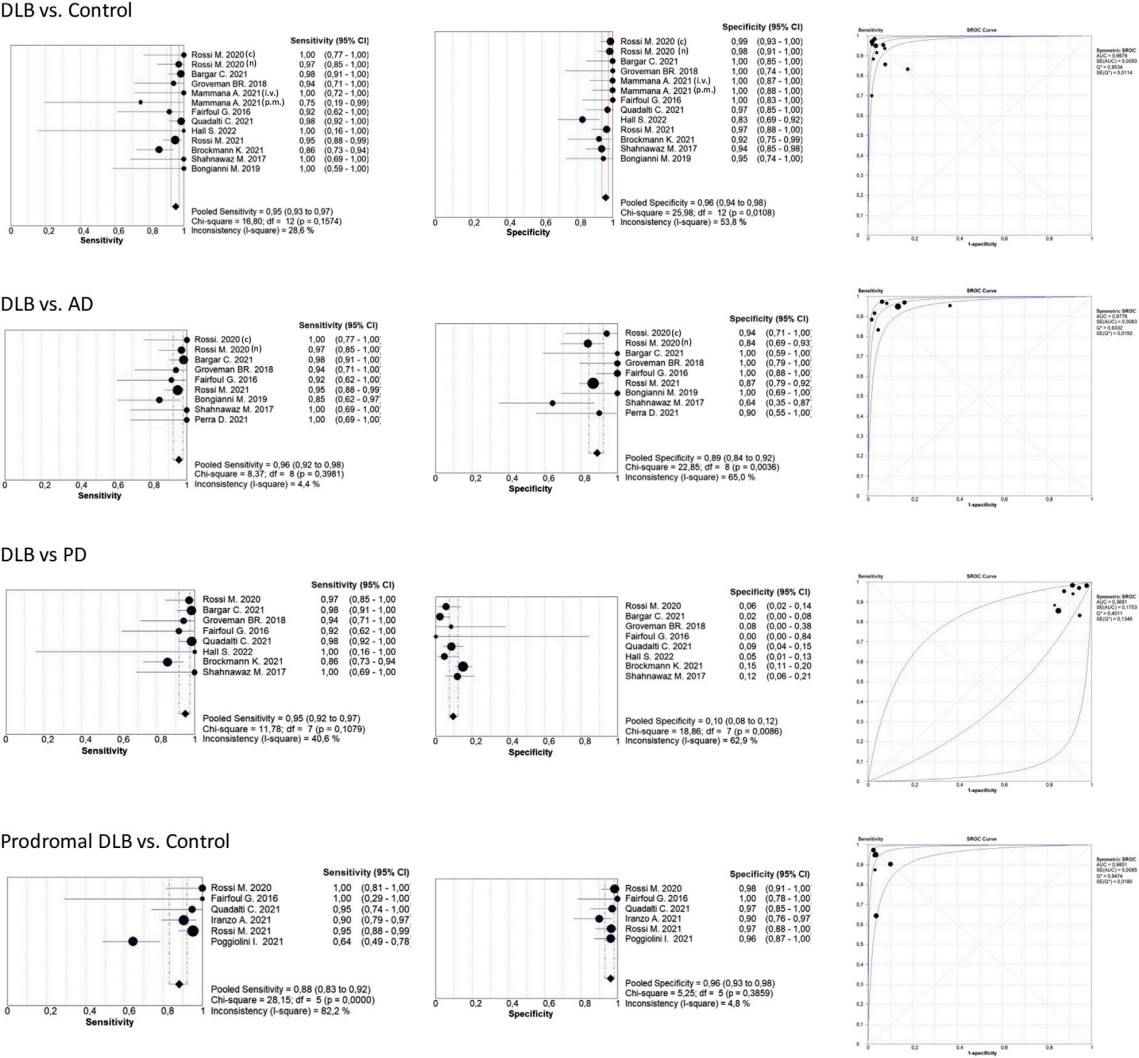
Forest plots meta-analysis CSF samples. The references that appear duplicated provided independent data for type of diagnosis or other conditions. These references are differentiated as follows: i. v. *In vitam*; p.m: post-mortem (*n*) not neuropathologically confirmed; (c) neuropathologically confirmed. For more detailed information see Tables 2, 3, 4, and 6.

Table 2 shows that most of the studies reported sensitivities and specificities above 90% in the differentiation between DLB and controls. Our meta-analysis showed that the pooled sensitivity and specificity are 0.94 (95% CI = 0.92,0.96) and 0.96 (0.95,0.98), respectively, including all sample types (Figure 3); and 0.95 (0.93,0.97) and 0.96 (0.94,0.98) for CSF samples (see Table 7; Figure 4). Two studies showed a sensitivity lower than 80%, which could likely be attributed to the small sample size or the specific brain area analysed in studies of brain tissue homogenates (Candelise et al., 2019; Mammana et al., 2021). In particular, the study carried out in samples from the frontal cortex and substance nigra showed the lowest sensitivity (70%) among all studies comparing DLB and controls (Candelise et al., 2019). The authors reported the existence of different α-syn strains (Candelise et al., 2019). Therefore, different α-syn strains may be differentially expressed in different brain areas, which could lead to a different performance of RT-QuIC depending on brain region.

**TABLE 2:** α-syn RT-QuIC results for DLB versus controls.

	Diagnostic groups	Neuropathologically confirmed	Sample type	TP	TN	FP	FN	Sensitivity	CI	Specificity	CI	Proportion of well-classified
Rossi et al. (2020)	DLB (n = 14)	Yes	CSF	14	80	1	0	100	78.5-100	98.8	93.3-99.8	0.99
	Non-α-syn control (n = 81)											
Rossi et al. (2020)	DLB (n = 34)	No	CSF	33	61	1	1	97.1	85.1-99.5	98.4	91.4-99.7	0.98
	Control (n = 62)											
Bargar et al. (2021)	DLB (n = 58)	Yes	CSF	57	23	0	1	98.3	90.9-99.7	100	85.7-100	0.99
	Control (n = 23)											
Bargar et al. (2021)	DLB (n = 3)	Yes	Brain	3	3	0	0	100	43.8-100	100	43.8-100	1
	Control (n = 3)											
Groveman et al. (2018)	DLB (n = 17)	No	CSF	16	12	0	1	94.1	73-99	100	75.7-100	0.97
	Control (n = 12)											
Mammana et al. (2021)	DLB (n = 11)	No	CSF (in vitam)	11	27	0	0	100	74.1-100	100	87.5-100	1
	Control (n = 27)											
Mammana et al. (2021)	DLB (n = 15)	No	Skin (in vitam)	15	39	2	0	100	79.6-100	95.1	83.9-98.7	0.96
	Control (n = 41)											
Mammana et al. (2021)	DLB (n = 4)	Yes	CSF (*postmortem*)	3	30	0	1	75	30.1-95.4	100	88.6-100	0.97
	Non-LBD (n = 30)											
Mammana et al. (2021)	DLB (n = 7)	Yes	Skin (*postmortem*)	6	39	1	1	85.7	48.7-97.4	97.5	87.1-99.6	0.96
	Non-LBD (n = 40)											
Han et al. (2020)	DLB (n = 7)	No	Brain	7	5	1	0	100	64.6-100	83.3	43.6-97	0.92
	Control (n = 6)											
Fairfoul et al. (2016)	DLB (n = 12)	Yes	CSF	11	20	0	1	91.7	64.6-98.5	100	83.9-100	1
	Control (n = 20)											
Wang et al. (2021)	DLB (n = 7)	No	Skin	7	42	1	0	100	64.6-100	97.7	87.9-99.6	0.98
	Control (N = 43)											
Quadalti et al. (2021)	DLB (n = 64)	No	CSF	63	34	1	1	98.4	91.7-99.7	97.1	85.5-99.5	0.98
	Control (N = 35)											
Hall et al. (2022)	DLB (n = 2)	Yes	CSF	2	39	8	0	100	34.2-100	83	69.9-91.1	0.84
	Control (n = 47)											
Sano et al. (2017)	DLB (n = 7)	Yes	Brain	6	2	0	1	85.7	48.7-97.4	100	34.2-100	0.89
	Control (n = 2)											
Rossi et al. (2021)	DLB (n = 81)	Partially	CSF	77	56	2	4	95.1	88-98.1	96.6	88.3-99	0.96
	Control (n = 58)											
Manne et al. (2019)	DLB (n = 5)	Yes	Brain	5	11	0	0	100	56.6-100	100	74.1-100	1
	Control (n = 11)											
Brockmann et al. (2021)	DLB (n = 49)	No	CSF	42	24	2	7	85.7	73.3-92.9	92.3	75.9-97.9	0.88
	Control (N = 26)											
Candelise et al. (2019)	DLB (n = 10)	Yes	Brain (Frontal cortex)	7	10	0	3	70	39.7-89.2	100	72.2-100	0.85
	Control (n = 10)											
Candelise et al. (2019)	DLB (n = 8)	Yes	Brain (Sust nigra)	6	5	0	2	75	40.9-92.9	100	56.6-100	0.85
	Control (n = 5)											
Shin et al. (2022)	DLB (n = 1)	Yes	Brain	1	2	0	0	100	20.7-100	100	34.2-100	1
	Control (n = 2)											
Shahnawaz et al. (2017)	DLB (n = 10)	No	CSF	10	61	4	0	100	72.2-100	93.8	85.2-97.6	0.95
	Neurological diseases (n = 65)											
Bongianni et al. (2019)	DLB (n = 7)	Yes	CSF	7	18	1	0	100	64.6-100	94.7	75.4-99.1	0.96
	Neurological diseases (n = 19)											

Abbreviations: DLB: Dementia with Lewy Bodies. CSF: cerebrospinal fluid.

Control groups: Neurological diseases: participants with neurological diseases that are not α-synucleinopathies or AD; Non-α-syn controls: participants with diseases other than α-synucleinopathies; Non-LBD: participants without LBD.

Partially: A subgroup of participants has neuropathological confirmation.

We compared sensitivity and specificity values by sample type [CSF (*n* = 13) vs brain (*n* = 7)] and type of diagnosis [clinical (*n* = 9) vs neuropathological (*n* = 13)]. We found no statistically significant differences in diagnostic performance depending on sample type (sensitivity *p* = 0.938, specificity *p* = 0.157) or type of diagnosis (sensitivity *p* = 0.357, but with a trend towards statistical significance in specificity *p* = 0.060, suggesting higher α-syn RT-QuIC specificities in individuals with a neuropathological diagnosis). Additionally, CSF vs other samples (combining brain and skin) did not show statistically significant differences in sensitivity (*p* = 0.927) and specificity (*p* = 0.376) values.

#### 3.2.2 DLB versus AD

Table 3 shows the comparison between DLB and AD groups. Most of the studies reported sensitivities and specificities above 90%. Our meta-analysis showed that the pooled sensitivity and specificity values are 0.95 (0.92,0.97) and 0.88 (0.84,0.92), respectively, including all sample types (Figure 3); and 0.96 (0.92,0.98) and 0.89 (0.84,0.92) for CSF samples (see Figure 4; Table 7).

**TABLE 3:** α-syn RT-QuIC results for DLB versus AD.

References number	Diagnostic groups	Neuropathologically confirmed	Sample type	TP	TN	FP	FN	Sensitivity	CI	Specificity	CI	Proportion of well clasified
Rossi et al. (2020)	DLB (n = 14)	Yes	CSF	14	16	1	0	100	78.5-100	94.1	73-99	0.97
	AD (n = 17)											
Rossi et al. (2020)	DLB (n = 34)	No	CSF	33	36	7	1	97.1	85.1-99.5	83.7	70-91.9	0.90
	AD (n = 43)											
Bargar et al. (2021)	DLB (n = 58)	Yes	CSF	57	7	0	1	98.3	90.9-99.7	100	64.6-100	0.98
	AD (n = 7)											
Groveman et al. (2018)	DLB (n = 17)	No	CSF	16	16	0	1	94.1	73-99	100	80.6-100	0.97
	AD (n = 16)											
Groveman et al. (2018)	DLB (n = 11)	Yes	Brain	11	5	0	0	100	74.1-100	100	56.6-100	1
	AD (n = 5)											
Fairfoul et al. (2016)	DLB (n = 12)	Yes	CSF	11	30	0	1	91.7	64.6-98.5	100	88.6-100	1
	AD (n = 30)											
Wang et al. (2021)	DLB (n = 7)	No	Skin	7	12	5	0	100	64.6-100	70.6	46.9-86.7	0.79
	AD (N = 17)											
Sano et al. (2017)	DLB (n = 7)	Yes	Brain	6	2	0	1	85.7	48.7-97.4	100	34.2-100	0.89
	AD (n = 2)											
Rossi et al. (2021)	DLB (n = 81)	Partially	CSF	77	104	16	4	95.1	88-98.1	86.7	79.4-91.6	0.84
	AD (n = 120)											
Bongianni et al. (2019)	DLB (n = 20)	No	CSF	17	10	0	3	85	64-94.8	100	72.2-100	0.90
	AD (n = 10)*											
Bongianni et al. (2019)	DLB (n = 3)	Yes	Brain	3	2	0	0	100	43.8-100	100	34.2-100	1
	AD (n = 2)											
Manne et al. (2019)	DLB (n = 5)	Yes	Brain	5	10	0	0	100	56.6-100	100	72.2-100	1
	AD (N = 10)											
Shahnawaz et al. (2017)	DLB (n = 10)	No	CSF	10	9	5	0	100	72.2-100	64.3	38.8-83.7	0.79
	AD (n = 14)											
Perra et al. (2021)	DLB (n = 32)	No	OM	27	9	1	5	84.4	68.2-93.1	90	59.6-98.2	0.86
	AD (n = 10)											
Perra et al. (2021)	DLB (n = 10)	No	CSF	10	9	1	0	100	72.2-100	90	59.6-98.2	0.95
	AD (n = 10)											

DLB: dementia with lewy bodies, AD: alzheimer disease, CSF: cerebrospinal fluid; MCI: mild cognitive impairment.

Partially: A subgroup of participants has neuropathological confirmation.

We found no statistically significant differences in sensitivity (*p* = 0.689) and specificity (*p* = 0.529) values when comparing sample types (CSF (*n* = 9) vs other sample types combined: brain (*n* = 4), skin (*n* = 1, OM (*n* = 1). In contrast, we found that the diagnostic capacity of α-syn RT-QuIC did depend on the type of diagnosis (clinical (*n* = 7) vs neuropathological (*n* = 7): sensitivity *p* = 0.535, specificity *p* = 0.038). Most of the studies with neuropathological confirmation showed 100% specificity, while the studies with clinical diagnoses showed specificities ranging from 64% to 100%.

#### 3.2.3 DLB versus PD

When comparing DLB versus PD (Table 4), our meta-analysis showed a pooled sensitivity of 0.94 (0.91,0.97) including all sample types (Figure 3) and 0.95 (0.92,0.97) for CSF samples (see Figure 4; Table 7). In contrast, our meta-analysis showed an extremely low pooled specificity of 0.11 (0.09,0.14) for all sample types; and 0.10 (0.08,0.12) for CSF samples. An exception is Candelise et al., who reported high specificities (90%-100%) with sensitivities around 70% when they analysed specific brain areas (frontal cortex and substantia nigra).

**TABLE 4:** α-syn RT-QuIC results for DLB versus PD.

References number	Diagnostic groups	Neuropathologically confirmed	Sample type	TP	TN	FP	FN	Sensitivity	CI	Specificity	CI	Proportion of well clasified
Rossi et al. (2020)	DLB (n = 34)	No	CSF	33	4	67	1	97.1	85.1-99.5	5.6	2.2-13.6	0.35
	PD (n = 71)											
Bargar et al. (2021)	DLB (n = 58)	Yes	CSF	57	2	86	1	98.3	90.9-99.7	2.3	0.6-7.9	0.40
	PD (n = 88)											
Bargar et al. (2021)	DLB (n = 3)	Yes	Brain	3	0	3	0	100	43.8-100	0	0-56.2	0.50
	PD (n = 3)											
Groveman et al. (2018)	DLB (n = 17)	No	CSF	16	1	11	1	94.1	73-99	8.3	1.5-35.4	0.59
	PD (n = 12)											
Mammana et al. (2021)	DLB (n = 11)	Partially	CSF	11	0	7	0	100	74.1-100	0	0-35.4	0.61
	PD (n = 7)											
Mammana et al. (2021)	DLB (n = 15)	Partially	Skin	15	3	10	0	100	79.6-100	23.1	8.2-50.3	0.64
	PD (n = 13)											
Fairfoul et al. (2016)	DLB (n = 12)	Yes	CSF	11	0	2	1	91.7	64.6-98.5	0	0-65.8	0.85
	PD (n = 2)											
Wang et al. (2021)	DLB (n = 7)	No	Skin	7	3	44	0	100	64.6-100	6.4	2.2-17.2	0.19
	PD (N = 47)											
Quadalti et al. (2021)	DLB (n = 64)	No	CSF	63	10	106	1	98.4	91.7-99.7	7.2	4.1-12.5	0.41
	PD (N = 116)											
Hall et al. (2022)	DLB (n = 2)	Yes	CSF	2	3	47	0	100	34.2-100	4.7	1.6-12.9	0.08
	PD (n = 50)											
Bongianni et al. (2019)	DLB (n = 3)	Yes	Brain	3	0	2	0	100	43.8-100	0	0-65.8	0.60
	PD sust nigra (n = 2)											
Manne et al. (2019)	DLB (n = 5)	Yes	Brain	5	1	10	0	100	56.6-100	9.1	1.6-37.7	0.38
	PD (N = 11)											
Brockmann et al. (2021)	DLB (n = 49)	No	CSF	42	35	200	7	85.7	73.3-92.9	14.9	10.9-20	0.27
	PD (n = 235)											
Candelise et al. (2019)	DLB (n = 10)	Yes	Brain (frontal cortex)	7	9	1	3	70	39.7-89.2	90	59.6-98.2	0.80
	PD (n = 10)											
Candelise et al. (2019)	DLB (n = 8)	Yes	Brain (sust nigra)	6	6	0	2	75	40.9-92.9	100	61-100	0.86
	PD (n = 6)											
Shin et al. (2022)	DLB (n = 1)	Yes	Brain	1	0	2	0	100	20.7-100	0	0-65.8	0.33
	PD (n = 2)											
Shahnawaz et al. (2017)	DLB (n = 10)	No	CSF	10	9	67	0	100	72.2-100	11.8	6.4-21	0.22
	PD (n = 76)											

Partially: A subgroup of participants has neuropathological confirmation.

In addition, we did not find any statistical significance in sensitivity and specificity values when comparing sample types [CSF (*n* = 9) vs brain (*n* = 6) (*p* = 0.776, *p* = 1)] or type of diagnosis [clinical (*n* = 6) vs neuropathological (*n* = 9)] (*p* = 0.864, *p* = 0.224). We neither obtained statistically significant differences in sensitivities and specificities when comparing CSF (*n* = 9) vs other sample types combined [brain (*n* = 6), skin (*n* = 2) (*p* = 0.423, *p* = 0.606)].

#### 3.2.4 DLB without co-pathologies versus DLB with co-pathologies

Table 5 summarizes the results on the α-syn RT-QuIC’s capacity to detect seeding activity in DLB groups without versus with co-pathologies. In pure DLB (cases without co-pathologies), the sensitivity was almost 100% for both CSF and brain tissue, while sensitivity values were lower (86%) for OM. In DLB cases with AD co-pathology, sensitivity was almost 100% (note: number of participants between 7-47) for both CSF and brain tissue, except for the study from Fairfoul et al., which reported a sensitivity value of 65% (11/17). However, in one study carried out in OM, sensitivity was only around 50% (3/6) when DLB cases had AD co-pathology (Perra et al., 2021). In another study, 15% (2/13) of AD cases with incidental LB showed positivity in α-syn RT-QuIC in CSF, and 0% (0/30) of the participants with pure AD were α-syn RT-QuIC positive (Fairfoul et al., 2016). In addition, Jin et al. reported the influence of the *APOE* ε4 allele on α-syn RT-QuIC results. They found that the intensity of ThT detected in the α-syn RT-QuIC assay was statistically significantly higher in *APOE* ε4 carriers in both groups (AD and DLB with AD co-pathology).

**TABLE 5:** α-syn RT-QuIC results for DLB without versus with co-pathologies.

	References/positive results in α-syn RT-QuIC						
	Groveman et al. (2018)	Fairfoul et al. (2016)	Hall et al. (2022)	Bongianni et al. (2019)	Jin et al. (2022)	Perra et al. (2021)	Perra et al. (2021)
Neuropathologically confirmed	Yes	Yes	Yes	Yes	Yes	No	No
Sample	Brain	CSF	CSF	CSF	Brain	OM	CSF
Pure DLB	100% (4/4)	92% (11/12)		100% (7/7)		86% (32/37)	100% (10/10)
DLB + AD	100% (7/7)	65% (11/17)	100% (25/25)	93% (14/15)	96% (45/47)	50% (3/6)	100% (6/6)
AD with incidental LB		15% (2/13)					
AD	0% (0/5)	0% (0/30)			44% (19/43)	10% (1/10)	10% (1/10)
LBS-PSP			0% (0/1)				
LBS-VaD			0% (0/2)				
LBS-VaD-PSP			100% (1/1)				
LBS-Astrocytome			100% (1/1)				
LBD/PART				100% (2/2)			
CJD/LBD				67% (2/3)			

DLB: dementia with lewy bodies; AD: alzheimer disease; LBS-PSP: lewy bodies; Progressive supranuclear palsy; VaD: vascular dementia; PART: Primary age-related tauopathy; CJD: Creutzfeldt-Jakob Disease.

In general, patients with pure AD showed negative α-syn RT-QuIC results in most cases, while one study showed α-syn RT-QuIC positive results in 44% (19/43) of the AD participants while most DLB patients with AD co-pathology [96% (45/47)] showed positive α-syn RT-QuIC results (Jin et al., 2022).

We also identified several studies investigating co-pathologies other than AD. In particular, the study by Hall et al. reported a negative result for α-syn RT-QuIC in 1/1 DLB cases with concomitant progressive supranuclear palsy (PSP) and 2/2 DLB cases with cerebrovascular co-pathology. In contrast, 1/1 DLB cases with concomitant cerebrovascular and PSP, and 1/1 DLB cases with concomitant astrocytome showed a positive α-syn RT-QuIC result. Bongianni et al. reported that 2/2 and 2/3 cases with Lewy body disorders (LBD) with concomitant primary age-related tauopathy and LBD with Creutzfeldt-Jakob disease were positive on α-syn RT-QuIC (Bongianni et al., 2019). Finally, Sakurai et al. did not find an influence of concomitant idiopathic normal pressure hydrocephalus on α-syn RT-QuIC results in patients with DLB (Sakurai et al., 2022).

#### 3.2.5 Prodromal DLB stages

Our meta-analysis for the detection of seeding activity in the prodromal DLB stages of iRBD and MCI-LB compared to controls (Table 6) showed a pooled sensitivity of 0.78 (0.73,0.83) and specificity of 0.95 (0.92,0.97) when including all the studies in this review (see Figure 3; Table 7). All these studies except for one were carried out on CSF samples. When performing the meta-analysis only on the studies including CSF samples, we obtained a pooled sensitivity of 0.88 (0.83,0.92) and specificity of 0.96 (0.93,0.98) (Figure 4). Comparisons for sample type and diagnosis type were not possible due to the small number of studies available.

**Table 6:** α-syn RT-QuIC results for prodromal DLB (iRBD, MCI-LB) versus controls.

References number	Diagnostic groups	Neuropathologically confirmed	Sample type	TP	TN	FP	FN	Sensitivity	CI	Specificity	CI	Proportion of well clasified
Rossi et al. (2020)	Control (n = 62)	No	CSF	18	61	1	0	100	82.4-100	98.4	91.4-99.7	0.99
	iRBD (n = 18)											
Fairfoul et al. (2016)	Control (n = 15)	Yes	CSF	3	15	0	0	100	43.8-100	100	79.6-100	1
	iRBD (n = 3)											
Quadalti et al. (2021)	Control (n = 35)	No	CSF	18	34	1	1	94.7	75.4-99.1	97.1	85.5-99.5	0.96
	iRBD (N = 19)											
Iranzo et al. (2021)	Control (n = 40)	No	CSF	47	36	4	5	90.4	79.4-95.8	90	76.9-96	0.90
	iRBD patients (n = 52)											
Rossi et al. (2021)	Control (n = 58)	Partially	CSF	77	56	2	4	95.1	88.1-98.1	96.6	88.3-99	0.96
	Probable MCI- LB (n = 81)											
Stefani et al. (2021)	Control (n = 59)	No	OM	28	53	6	35	44.4	32.8-56.7	89.8	79.5-95.3	0.66
	iRBD (n = 63)											
Poggiolini et al. (2022)	Control (n = 55)	No	CSF	29	53	2	16	64.4	49.8-76.8	96.4	87.7-99	0.82
	iRBD (n = 45)											

iRBD: isolated rapid eye movement sleep behavior disorder.

Partially: A subgroup of participants has neuropathological confirmation.

**TABLE 7:** Meta-analytical estimates.

	All sample types			CSF		
	Sensitivity (%, CI)	Specificity (%, CI)	sROC	Sensitivity (%, CI)	Specificity (%, CI)	sROC
DLB vs. Control	0.94 (0.92-0.96)	0.96 (0.95-0.98)	0.98	0.95 (0.93-0.97)	0.96 (0.94-0.98)	0.99
DLB vs. AD	0.95 (0.92-0.97)	0.88 (0.84-0.92)	0.97	0.96 (0.92-0.98)	0.89 (0.84-0.92)	0.97
DLB vs. PD	0.94 (0.91-0.97)	0.11 (0.09-0.14)	0.78	0.95 (0.92-0.97)	0.10 (0.08-0.12)	0.3691
Prodromal DLB vs. Control	0.78 (0.73-0.83)	0.95 (0.92-0.97)	0.99	0.88 (0.83-0.92)	0.96 (0.93-0.98)	0.99

sROC, summary receiver operating characteristic.

CI, confidence interval.

Iranzo et al. reported a sensitivity value of 90% for iRBD. In their longitudinal analyses, the authors reported a sensitivity value of 100% for iRBD cases that phenoconverted to DLB and 94% for iRBD cases that phenoconverted to PD (Iranzo et al., 2021). The sensitivity value in iRBD cases that remained stable (did not phenoconvert to DLB or PD) was 80%. Rossi et al. reported a sensitivity value of 95% in MCI-LB when compared with normal controls (Rossi et al., 2021). In all studies with prodromal DLB stages, the specificities were also at least 90%.

#### 3.2.6 Neuropathological DLB subtypes

Two studies investigated whether α-syn RT-QuIC performs differently across neuropathological subtypes of DLB. Specifically, Hall et al. reported that α-syn RT-QuIC’s performance in CSF samples is different in DLB patients with cortical pathology as compared with brainstem/amygdala pathology. The CSF test was positive for 97% of DLB patients with cortical pathology while it was positive in 50% of DLB patients with brainstem/amygdala pathology. Specifically, 0/2 DLB patients with brainstem pathology, 5/7 DLB patients with amygdala pathology and, 2/5 DLB patients with pathology in both brainstem and amygdala showed positive α-syn RT-QuIC results (Hall et al., 2022). Sano et al. showed that 100% (6/6) of DLB patients with cortical pathology had a positive α-syn RT-QuIC result, while the only one patient with limbic pathology (0/1) had a negative α-syn RT-QuIC result (Sano et al., 2017).

#### 3.2.7 Genetic variants

Specific genetic variants have also been suggested to be a source of potential variability in the diagnostic performance of α-syn RT-QuIC. Brockmann et al. reported that 100% of the DLB patients with the GBA mutation showed positivity in α-syn RT-QuIC, while 79% of non-GBA carriers DLB patients were positive in α-syn RT-QuIC (Brockmann et al., 2021). The authors suggested that certain genetic variants may generate a more intense pathology, which can be seen histopathologically. Specifically, patients with GBA mutations showed greater Lewy body pathology histologically. That more intense pathology in GBA mutations could thus explain the higher sensitivity of the RT-QuIC assay. In addition, as mentioned above, the seeding activity (maximum ThT fluorescence) is higher in patients with AD and concomitant LBD who are *APOE* ε4 carriers, as compared with *APOE* ε4 non-carries (Jin et al., 2022). An explanation for this finding is that *APOE* ε4 aggregates could promote a conformational change of α-syn which could result in a higher seeding activity (Jin et al., 2022).

### 3.3 The influence of variability on experimental conditions on diagnostic performance of α-syn RT-QuIC in DLB

After inspecting the reviewed studies, we observed differences in the conditions used for the α-syn RT-QuIC analysis (see Table 1). In general, the different technical conditions (incubation conditions, sample volume, recombinant α-syn, beads, salt concentrations, and buffer) did not show overt differences in the diagnostic performance of α-syn RT-QuIC between DLB and control groups.

Specifically, for DLB vs control groups, most of the studies fixed the temperature at 42°C (*n* = 16), while some studies fixed the temperature at 30°C (*n* = 1), 37°C (*n* = 5) or 40°C (*n* = 1) (Table 1). When we tested for statistical differences between studies using different temperatures [42°C (*n* = 16) vs other temperatures combined (*n* = 7)], we did not find any effect of temperature in sensitivity (*p* = 0.890) or specificity (*p* = 0.154) values. Regarding the time of shaking-rest cycles, 1 min shaking - 1 min rest is the most used setup (*n* = 18), while some studies used 1 min shaking—14 min rest (*n* = 1), 1 min shaking—29 min rest (*n* = 3) or 40 s shaking—140s rest (*n* = 1). We found no statistically significant differences in sensitivity (*p* = 0.403) and specificity (*p* = 0.130) values when comparing studies with 1 min shaking-1 min rest (*n* = 18) vs the studies using other shaking-rest cycles combined (*n* = 5). Similarly, we observed that studies using different shaking speeds [400 rpm (*n* = 18), 432 rpm (*n* = 1), 500 rpm (*n* = 1), 600 rpm (*n* = 1), and 200 rpm (*n* = 1)] did not differ statistically in the reported sensitivity (*p* = 0.403) and specificity (*p* = 0.130) values when comparing 400 rpm vs the other speed conditions combined. Regarding the sample volume, almost all studies used 15 µL for CSF samples while 2 µL is the most common for brain samples. We could not analyse statistically the influence of sample volume on α-syn RT-QuIC results due to the small number of studies using alternative sample volumes.

Regarding the reaction mix, we assessed NaCl concentration, SDS concentration, buffer concentration, and buffer pH (please see section “*2.3. Outcome measures and statistical analysis*” for a description of the studies and categories compared). None of these conditions showed differences in sensitivity or specificity values when comparing the most common condition vs the others conditions combined: NaCl concentration [170 mM (*n* = 18) vs 160 Mm (*n* = 1), 500 mM (*n* = 1), and without NaCl (*n* = 3) combined]: sensitivity *p* = 0.431, specificity *p* = 0.658; SDS concentration [0.0015% (*n* = 14) vs 0.00125% (*n* = 2), 0.005% (*n* = 2), and without SDS (*n* = 5) combined]: sensitivity *p* = 0.691, specificity *p* = 0.999; buffer concentration [40 mM (*n* = 16) vs other concentrations combined (*n* = 7)]: sensitivity *p* = 0.341, specificity *p* = 0.452; and buffer pH [8 (*n* = 16), other pHs combined (*n* = 7)]: sensitivity *p* = 0.341, specificity *p* = 0.452.

In addition, the recombinant α-syn may also be an important factor in the assay. Most of the studies included in this review used a wild-type α-syn or the α-syn mutant K23Q (Table 1). The single amino acid mutant K23Q produces kinetics similar to wild type when seeded with α-syn fibrils, but its spontaneous aggregation is lower without seeding than that in wild type. This property of the mutant results in higher sensitivity of the assay (Koo et al., 2008; Groveman et al., 2018). Moreover, some studies used a commercial α-syn, while other studies used an in-house protein (Table 1). Sensitivity and specificity values did not show statistically significant differences when comparing commercial and in-house α-syn (*p* = 0.671, *p* = 0.249). However, the differences between the commercial and in-house α-syn are difficult to assess due to the high variability across studies.

## 4 Discussion

In this systematic review, we assessed the diagnostic performance of α-syn seeding activity detection by RT-QuIC in DLB. Overall, we found that α-syn RT-QuIC can discriminate DLB patients from AD and control groups well, while it cannot discriminate between DLB and PD patients at the moment. Moreover, we observed a promising α-syn RT-QuIC performance in prodromal DLB stages. Preliminary data suggest that α-syn RT-QuIC may have a higher diagnostic performance in DLB patients with diffuse neocortical pathology, and who are carriers of GBA mutations or the *APOE* ε4 allele (Jin et al., 2022). We also analysed whether α-syn RT-QuIC’s performance differs depending on factors such as the sample type, the type of diagnosis, the different parameters of the RT-QuIC assay, and variations in experimental conditions.

We observed that α-syn RT-QuIC is useful for DLB diagnosis, particularly for discriminating DLB patients from non-α-syn groups like controls and AD patients. While this high performance was known, our current study provides the meta-analytical estimates of 0.94 and 0.95 sensitivity and 0.96 and 0.88 specificity, respectively for controls and AD patients. Our meta-analytical values based on 23 and 15 studies are a robust piece of evidence towards α-syn RT-QuIC successfully passing the threshold of 80% accuracy for a measure to be considered a diagnostic biomarker (The Ronald and Nancy Reagan Research Institute of the Alzheimer’s Association and National Institute on Aging Working GroupAB, 1998). Therefore, our meta-analytical results encourage to consider the setup of α-syn RT-QuIC for clinical use in DLB. A recent publication showed a high sensitivity of α-syn seed amplification assays in a large cohort for PD patients, thus highlighting the clinical potential of α-syn aggregation detection for PD as well (Siderowf et al., 2023). Further, our statistical analyses suggest that α-syn RT-QuIC’s diagnostic performance seems comparable across different samples. Therefore, CSF could be considered as a good diagnostic option, since it is a relatively accessible sample. However, in cases where the lumbar puncture is contraindicated, skin biopsy and OM samples may serve as a good alternative thanks to their fair accuracy (Manne et al., 2020; Mammana et al., 2021; Perra et al., 2021). However, more studies are required to confirm the first skin biopsy and OM reports.

Even though α-syn RT-QuIC discriminates DLB patients from controls and AD patients very well, we observed slight differences. α-syn RT-QuIC’s ability to discriminate DLB from AD individuals was slightly lower than when discriminating DLB from controls. It has been discussed that the frequent DLB-AD co-pathology may explain the slightly lower discrimination between DLB and AD. This may be particularly true or mostly influenced by results from studies without neuropathological confirmation. However, this literature is still increasing and in our systematic review, we observed that the influence of co-pathologies in α-syn RT-QuiC’s performance is still poorly understood. Some studies reported an impact of AD co-pathology in α-syn RT-QuiC results while other studies did not. We suggest that future α-syn RT-QuIC studies in DLB try to report results stratifying by AD co-pathology if possible. This may also have implications for clinical trials, as recently discussed by Toledo et al. (2022). The finding of positive α-syn RT-QuIC results in AD patients with LB co-pathology further cross-validates and supports this suggestion (Rossi et al., 2021). Two studies reported results on co-pathologies other than AD (Bongianni et al., 2019; Hall et al., 2022), but the small sample sizes did not allow us to perform any statistical analysis or draw conclusions in the context of our review. We thus encourage that future studies investigate the potential effect of other co-pathologies on α-syn RT-QuIC in DLB.

We also observed a low capacity of α-syn RT-QuIC to discriminate between DLB and PD, as expected since both are α-synucleinopathies. However, the lack of discriminatory ability between DLB and PD may be because previous studies mainly limited their analysis to the dichotomous outcome of the α-syn RT-QuIC assay (positive vs negative). Recent data suggests that perhaps specific parameters of the seeding activity could be useful to detect differences and discriminate DLB from PD. For example, Rossi et al. reported a slight trend towards higher maximum intensities and area under the curve (AUC) in DLB patients than in PD (Rossi et al., 2020). In another study, Bargar et al. found higher global seeding activities and shorter lag phase in DLB than in PD, in reactions seeded by 0.02–2 μL CSF; and the protein aggregation rate was significantly higher in DLB than in PD. The authors also found that PD cases displayed positive α-syn RT-QuIC responses within ∼18–50 h, while DLB cases showed strong ThT responses within ∼15–30 h (Bargar et al., 2021). In addition, Groveman et al. also found differences in kinetics between PD and DLB perhaps related to different seeding concentrations or differences in the structures of seeds between the two groups (Groveman et al., 2018). Poggiolini et al. found high sensitivity (88%) and specificity (100%) values in the differentiation between PD and DLB using the detergent-soluble fraction of frontal cortex homogenate and α-syn 1-130 as the seeding substrate (Poggiolini et al., 2021). Candelise et al. studied α-syn RT-QuIC end products by Western blot, dot blot analysis, Raman spectroscopy, atomic force microscopy, and transmission electron microscopy suggesting the possibility for the existence of different α-syn strains in DLB and PD (Candelise et al., 2019). Specifically, they found resistance to proteinase K treatment in α-syn RT-QuIC products from DLB patients but not in PD or control participants, by immunoblot. Also, the authors observed different kinetics of α-syn RT-QuIC in patients with DLB compared to PD and controls (Candelise et al., 2019). Therefore, future studies should continue elucidating the α-syn RT-QuIC parameters that can differentiate DLB from PD and reveal what they can tell us about pathophysiological differences between these two a-synucleinopathies, *in-vivo*.

Concerning prodromal DLB, some recent data indicates that RT-QuIC in CSF samples can detect α-syn pathology in prodromal DLB phases, suggesting its potential for the early diagnosis of DLB. The study by Rossi et al. found sensitivity and specificity values close to 100% when comparing MCI-LB and controls (Rossi et al., 2021). However, there are still some questions to be addressed, especially regarding iRBD cases. For example, Iranzo et al. (2021) reported that 80% of iRBD individuals who did not convert to PD or DLB within 7-9 years also showed positive α-syn RT-QuIC results. α-syn pathology in stable iRBD could in principle be possible, and longer follow-ups could show phenotypical conversion in those cases, or neuropathological assessment could confirm LB pathology. But until this is clarified, this finding suggests that α-syn RT-QuIC may play a role in the diagnosis of LB pathology in pre-dementia stages, but its prognostic value for clinical phenoconversion seems limited and still needs to be investigated. Future research needs to clarify whether misfolded α-syn by RT-QuIC will serve as a state biomarker, while other markers may better serve as stage markers. For example, recent data shows the potential of the neurofilament light chain (NFL) in plasma as a stage marker (Pilotto et al., 2021).

Our systematic review and analysis of experimental conditions for α-syn RT-QuIC in CSF revealed that most studies used 15 µL of the sample, often in a 40 mM PB, pH 8.0, with 170 mM NaCl and usually with 0.0015% SDS. Reading conditions were often at 42°C with intermittent double orbital shaking at 400 rpm for 1 minute followed by 1 min rest. In addition, it is common to use 6 silica beads (0.8 mm) in each well. However, several modifications have been tested as well. For example, Poggiolini et al. tested two different reaction buffers (100 mM piperazine-N,N′-bis (ethanesulfonic acid) (PIPES; pH 6.9) and 1X PBS (10 mM sodium phosphate, 138 mM NaCl, and 2.7 mM KCl; pH 7.4)) on PD samples. The authors demonstrated an increase in fluorescence during the reaction time only for the 100 mM piperazine-N,N′-bis (ethanesulfonic acid) (PIPES; pH 6.9) buffer (Poggiolini et al., 2021). In contrast, we observed that the analytical conditions are more variable in tissue samples. However, their impact on α-syn RT-QuIC results is difficult to assess because of the limited number of studies using some particular conditions and the low variability in tissues and brain areas used across studies. Sample volumes are quite similar across studies using the same sample type. However, the purity of the sample could be an influential factor that we could not analyse due to lack of sufficient reporting in primary studies. We observed that CSF samples are often used directly without any dilutions. However, in studies performed on tissue, sample pre-processing is variable across studies. Fairfoul et al. tested 3 different sample volumes (5, 10, and 15 µL) and the reported α-syn RT-QuIC results were similar (Fairfoul et al., 2016). In addition, Bargar et al. showed that CSF volumes from 2 to 0.002 μL rendered similar α-syn RT-QuIC results in terms of positivity in DLB and PD samples. However, the authors reported that the protein aggregation rate, increased from 0.02 to 0.2, and afterwards was stable or decreased in higher volumes, possibly due to inhibitors in the sample (Bargar et al., 2021). Regarding brain homogenate tissue, Manne et al. (2019) reported that the best reproducibility of results seems to be with 1:10000 dilutions (Manne et al., 2019).

When reviewing the α-syn RT-QuIC literature in DLB, we observed that several studies come from the same research groups. This comes with the advantage that the methods are rather consistent across studies, but we observed that variations in assay have also been carried out and tested. The different conditions used in the studies included in this review did not seem to influence the α-syn RT-QuIC results—the technique showed good sensitivities and specificities, particularly in the comparison between DLB and controls. However, we cannot completely exclude the possibility of certain publication bias: perhaps only those methodological variations that are successful and produce positive results reach publication and, hence, there is very small variability in the literature that prevented us to obtain significant differences across methodological variations in our statistical analyses. Further, several of our statistical analyses included a low number of studies, or different conditions were combined. Future studies should thus continue testing the potential influence of conditions on α-syn RT-QuIC’s performance. For example, more dedicated analyses like the one performed by Candelise et al., show that factors such as pH, temperature, shaking, or metal ions affect α-syn aggregation by increasing or inhibiting the process (Candelise et al., 2020). In our own experience, this could highlight the need for different labs to adjust the method to make it work locally. Therefore, there is a need for harmonization or multi-lab validation of α-syn RT-QuIC to advance in the establishment and implementation of this technique in clinical practice, as it has previously occurred with CSF biomarkers for AD (Vanderstichele et al., 2012)

Regarding α-syn recombinant, our statistical analyses showed that the use of commercial or in-house protein did not show an influence on α-syn RT-QuIC’s performance. However, there is wide variability among commercial proteins and even more among the in-house ones, which requires further analysis. Poggiolini et al. investigated different C-terminally truncated recombinant α-syn as a substrate in the RT-QuIC assay in PD and DLB brain lysates as seeds. The authors suggested that α-syn species could differentiate between PD and DLB using homogenates from different brain areas (Poggiolini et al., 2021). Although the data on MSA is currently more limited, a study reported negative α-syn RT-QuIC results in most of the MSA cases (Rossi et al., 2020). A possible explanation for this differential performance of α-syn RT-QuIC in MSA could be the known differences in α-syn structure between MSA and DLB/PD (Rossi et al., 2020).

This systematic review has some limitations. First, the number of studies included is relatively low for some sub-analyses because α-syn RT-QuIC is a rather new technique. This mostly affected statistical analyses in comparisons by sample type or α-syn RT-QuIC’s technical conditions. To minimize this problem, we grouped different sample types (brain, skin, OM) or compared the most common condition vs the other conditions combined. This should be considered when interpreting those particular results. The number of studies included and sample sizes are reflected in the tables and the text. In addition, although most studies recruited participants using similar diagnostic criteria, the inclusion criteria may vary slightly depending on the aims of the studies. This may lead to difficulties in comparing some results across studies, particularly with regard the control groups. Regarding the α-syn RT-QuiC assay, different conditions (incubation and shaking time, buffer composition, substrate protein, etc.) were used across studies. However, we assessed the effect of each variable on sensitivity and specificity values, which is one of the contributions of our current systematic review. Lastly, several studies come from the same research groups and only 11/25 studies without overlapping authors were detected by our systematic review. The availability of more data in the future will help assess the generalization of results, towards considering α-syn RT-QuIC for its clinical use. Multicentre validation of α-syn RT-QuIC is still required for its clinical application.

In conclusion, this systematic review and meta-analysis shows that misfolded α-syn detection by RT-QuIC has a good diagnostic capacity for the discrimination between DLB and controls, as well as for the differential diagnosis between DLB and AD. However, the dichotomous positive/negative α-syn RT-QuIC result is not able to discriminate DLB from PD, while future research will elucidate whether specific α-syn RT-QuIC parameters could help in that discrimination. The potential influence of co-pathologies in α-syn RT-QuIC results also needs to be further investigated. This review also reports the potential for RT-QuIC to detect α-syn at prodromal stages of DLB, but this finding needs to be confirmed neuropathologically and its prognostic value is to be determined. Finally, the technical conditions reported in the published studies do not seem to affect α-syn RT-QuIC’s performance. Nonetheless, the harmonization of protocols is essential to bring α-syn RT-QuIC to clinical practice.

## References

[B1] Bargar C. Wang W. Gunzler S. A. LeFevre A. Wang Z. Lerner A. J. (2021). Streamlined alpha-synuclein RT-QuIC assay for various biospecimens in Parkinson’s disease and dementia with Lewy bodies. Acta Neuropathol. Commun. 9 (1), 62. 10.1186/s40478-021-01175-w 33827706 PMC8028088

[B2] Bongianni M. Ladogana A. Capaldi S. Klotz S. Baiardi S. Cagnin A. (2019). α-Synuclein RT-QuIC assay in cerebrospinal fluid of patients with dementia with Lewy bodies. Ann. Clin. Transl. Neurol. 6 (10), 2120–2126. 10.1002/acn3.50897 31599499 PMC6801172

[B3] Brockmann K. Quadalti C. Lerche S. Rossi M. Wurster I. Baiardi S. (2021). Association between CSF alpha-synuclein seeding activity and genetic status in Parkinson’s disease and dementia with Lewy bodies. Acta Neuropathol. Commun. 9 (1), 175. 10.1186/s40478-021-01276-6 34717775 PMC8556894

[B4] Candelise N. Schmitz M. Llorens F. Villar‐Piqué A. Cramm M. Thom T. (2019). Seeding variability of different alpha synuclein strains in synucleinopathies. Ann. Neurol. 85 (5), 691–703. 10.1002/ana.25446 30805957

[B5] Candelise N. Schmitz M. Thüne K. Cramm M. Rabano A. Zafar S. (2020). Effect of the micro-environment on α-synuclein conversion and implication in seeded conversion assays. Transl. Neurodegener. 9 (1), 5. 10.1186/s40035-019-0181-9 31988747 PMC6966864

[B6] Colby D. W. Zhang Q. Wang S. Groth D. Legname G. Riesner D. (2007). Prion detection by an amyloid seeding assay. Proc. Natl. Acad. Sci. 104 (52), 20914–20919. 10.1073/pnas.0710152105 18096717 PMC2409241

[B7] Dong T.-T.-T. Satoh K. (2021). The latest research on RT-QuIC assays—a literature review. Pathogens 10 (3), 305. 10.3390/pathogens10030305 33807776 PMC8000803

[B8] Erkkinen M. G. Kim M.-O. Geschwind M. D. (2018). Clinical neurology and epidemiology of the major neurodegenerative diseases. Cold Spring Harb. Perspect. Biol. 10 (4), a033118. 10.1101/cshperspect.a033118 28716886 PMC5880171

[B9] Fairfoul G. McGuire L. I. Pal S. Ironside J. W. Neumann J. Christie S. (2016). Alpha-synuclein RT-QuIC in the CSF of patients with alpha-synucleinopathies. Ann. Clin. Transl. Neurol. 3 (10), 812–818. 10.1002/acn3.338 27752516 PMC5048391

[B10] Gomperts S. N. (2016). Lewy body dementias: Dementia with Lewy bodies and Parkinson disease dementia. CONTINUUM Lifelong Learn. Neurol. 22 (2), 435–463. 10.1212/CON.0000000000000309 PMC5390937 27042903

[B11] Groveman B. R. Orrù C. D. Hughson A. G. Raymond L. D. Zanusso G. Ghetti B. (2018). Rapid and ultra-sensitive quantitation of disease-associated α-synuclein seeds in brain and cerebrospinal fluid by αSyn RT-QuIC. Acta Neuropathol. Commun. 6 (1), 7. 10.1186/s40478-018-0508-2 29422107 PMC5806364

[B12] Hall S. Orrù C. D. Serrano G. E. Galasko D. Hughson A. G. Groveman B. R. (2022). Performance of αSynuclein RT-QuIC in relation to neuropathological staging of Lewy body disease. Acta Neuropathol. Commun. 10 (1), 90. 10.1186/s40478-022-01388-7 35733234 PMC9219141

[B13] Han J.-Y. Jang H.-S. Green A. J. E. Choi Y. P. (2020). RT-QuIC-based detection of alpha-synuclein seeding activity in brains of dementia with Lewy Body patients and of a transgenic mouse model of synucleinopathy. Prion 14 (1), 88–94. 10.1080/19336896.2020.1724608 32041499 PMC7039666

[B14] Heras-Garvin A. Stefanova N. (2020). From synaptic protein to prion: The long and controversial journey of α-synuclein. Front. Synaptic Neurosci. 12, 584536. 10.3389/fnsyn.2020.584536 33071772 PMC7536368

[B15] Iranzo A. Fairfoul G. Ayudhaya A. C. N. Serradell M. Gelpi E. Vilaseca I. (2021). Detection of α-synuclein in CSF by RT-QuIC in patients with isolated rapid-eye-movement sleep behaviour disorder: A longitudinal observational study. Lancet Neurol. 20 (3), 203–212. 10.1016/S1474-4422(20)30449-X 33609478

[B16] Jellinger K. A. Attems J. (2008). Prevalence and impact of vascular and Alzheimer pathologies in Lewy body disease. Acta Neuropathol. 115 (4), 427–436. 10.1007/s00401-008-0347-5 18273624

[B17] Jin Y. Li F. Sonoustoun B. Kondru N. C. Martens Y. A. Qiao W. (2022). APOE4 exacerbates α-synuclein seeding activity and contributes to neurotoxicity in Alzheimer’s disease with Lewy body pathology. Acta Neuropathol. 143 (6), 641–662. 10.1007/s00401-022-02421-8 35471463 PMC9107450

[B18] Koo H.-J. Lee H.-J. Im H. (2008). Sequence determinants regulating fibrillation of human alpha-synuclein. Biochem. Biophys. Res. Commun. 368 (3), 772–778. 10.1016/j.bbrc.2008.01.140 18261982

[B19] Kotzbauer P. T. Tu Z. Mach R. H. (2017). Current status of the development of PET radiotracers for imaging alpha synuclein aggregates in Lewy bodies and Lewy neurites. Clin. Transl. Imaging 5 (1), 3–14. 10.1007/s40336-016-0217-4

[B20] Kurapova R. Chouliaras L. O’Brien J. T. (2022). The promise of amplification assays for accurate early detection of α-synucleinopathies: A review. Exp. Gerontol. 165, 111842. 10.1016/j.exger.2022.111842 35623540

[B21] Mammana A. Baiardi S. Quadalti C. Rossi M. Donadio V. Capellari S. (2021). RT-QuIC detection of pathological α-synuclein in skin punches of patients with Lewy body disease. Mov. Disord. 36 (9), 2173–2177. 10.1002/mds.28651 34002890 PMC8518528

[B22] Manne S. Kondru N. Hepker M. Jin H. Anantharam V. Lewis M. (2019). Ultrasensitive detection of aggregated α-synuclein in glial cells, human cerebrospinal fluid, and brain tissue using the RT-QuIC assay: New high-throughput neuroimmune biomarker assay for parkinsonian disorders. J. Neuroimmune Pharmacol. 14 (3), 423–435. 10.1007/s11481-019-09835-4 30706414 PMC6669119

[B23] Manne S. Kondru N. Jin H. Anantharam V. Huang X. Kanthasamy A. (2020). α-Synuclein real-time quaking-induced conversion in the submandibular glands of Parkinson's disease patients. Mov. Disord. 35 (2), 268–278. 10.1002/mds.27907 31758740 PMC7102508

[B24] McKeith I. G. Boeve B. F. Dickson D. W. Halliday G. Taylor J.-P. Weintraub D. (2017). Diagnosis and management of dementia with Lewy bodies: Fourth consensus report of the DLB Consortium. Neurology 89 (1), 88–100. 10.1212/WNL.0000000000004058 28592453 PMC5496518

[B25] Morozova O. A. March Z. M. Robinson A. S. Colby D. W. (2013). Conformational features of tau fibrils from Alzheimer’s disease brain are faithfully propagated by unmodified recombinant protein. Biochemistry 52 (40), 6960–6967. 10.1021/bi400866w 24033133 PMC4142060

[B26] Nakagaki T. Nishida N. Satoh K. (2021). Development of α-synuclein real-time quaking-induced conversion as a diagnostic method for α-synucleinopathies. Front. Aging Neurosci. 13, 703984. 10.3389/fnagi.2021.703984 34650422 PMC8510559

[B27] Orru C. D. Wilham J. M. Vascellari S. Hughson A. G. Caughey B. (2012). New generation QuIC assays for prion seeding activity. Prion 6 (2), 147–152. 10.4161/pri.19430 22421206 PMC7082091

[B28] Perra D. Bongianni M. Novi G. Janes F. Bessi V. Capaldi S. (2021). Alpha-synuclein seeds in olfactory mucosa and cerebrospinal fluid of patients with dementia with Lewy bodies. Brain Commun. 3 (2), fcab045. 10.1093/braincomms/fcab045 33870192 PMC8042247

[B29] Pilotto A. Imarisio A. Carrarini C. Russo M. Masciocchi S. Gipponi S. (2021). Plasma neurofilament light chain predicts cognitive progression in prodromal and clinical dementia with Lewy bodies. J. Alzheimer’s Dis. 82 (3), 913–919. 10.3233/JAD-210342 34151807

[B30] Poggiolini I. Erskine D. Vaikath N. N. Ponraj J. Mansour S. Morris C. M. (2021). RT-QuIC using C-terminally truncated α-synuclein forms detects differences in seeding propensity of different brain regions from synucleinopathies. Biomolecules 11 (6), 820. 10.3390/biom11060820 34072869 PMC8226794

[B31] Poggiolini I. Gupta V. Lawton M. Lee S. El-Turabi A. Querejeta-Coma A. (2022). Diagnostic value of cerebrospinal fluid alpha-synuclein seed quantification in synucleinopathies. Brain 145 (2), 584–595. 10.1093/brain/awab431 34894214 PMC9014737

[B32] Quadalti C. Calandra-Buonaura G. Baiardi S. Mastrangelo A. Rossi M. Zenesini C. (2021). Neurofilament light chain and α-synuclein RT-QuIC as differential diagnostic biomarkers in parkinsonisms and related syndromes. Npj Parkinson’s Dis. 7 (1), 93. 10.1038/s41531-021-00232-4 34635674 PMC8505434

[B33] Rossi M. Candelise N. Baiardi S. Capellari S. Giannini G. Orrù C. D. (2020). Ultrasensitive RT-QuIC assay with high sensitivity and specificity for Lewy body-associated synucleinopathies. Acta Neuropathol. 140 (1), 49–62. 10.1007/s00401-020-02160-8 32342188 PMC7299922

[B34] Rossi M. Baiardi S. Teunissen C. E. Quadalti C. van de Beek M. Mammana A. (2021). Diagnostic value of the CSF α-synuclein real-time quaking-induced conversion assay at the prodromal MCI stage of dementia with Lewy bodies. Neurology 97 (9), e930–e940. 10.1212/WNL.0000000000012438 34210822 PMC8408510

[B35] Sakurai A. Tsunemi T. Ishiguro Y. Okuzumi A. Hatano T. Hattori N. (2022). Comorbid alpha synucleinopathies in idiopathic normal pressure hydrocephalus. J. Neurology 269 (4), 2022–2029. 10.1007/s00415-021-10778-1 34468800

[B36] Salvadores N. Shahnawaz M. Scarpini E. Tagliavini F. Soto C. (2014). Detection of misfolded aβ oligomers for sensitive biochemical diagnosis of Alzheimer’s disease. Cell Rep. 7 (1), 261–268. 10.1016/j.celrep.2014.02.031 24656814

[B37] Sano K. Atarashi R. Satoh K. Ishibashi D. Nakagaki T. Iwasaki Y. (2017). Prion-like seeding of misfolded α-synuclein in the brains of dementia with Lewy body patients in RT-QUIC. Mol. Neurobiol. 55, 3916–3930. 10.1007/s12035-017-0624-1 28550528 PMC5884914

[B38] Shahnawaz M. Tokuda T. Waragai M. Mendez N. Ishii R. Trenkwalder C. (2017). Development of a biochemical diagnosis of Parkinson disease by detection of α-synuclein misfolded aggregates in cerebrospinal fluid. JAMA Neurol. 74 (2), 163–172. 10.1001/jamaneurol.2016.4547 27918765

[B39] Shin C. Han J.-Y. Kim S.-I. Park S.-H. Yang H.-K. Lee H.-J. (2022). *In vivo* and autopsy validation of alpha-synuclein seeding activity using RT-QuIC assay in the gastrointestinal tract of patients with Parkinson’s disease. Park. Relat. Disord. 103, 23–28. 10.1016/j.parkreldis.2022.08.012 36029607

[B40] Siderowf A. Concha-Marambio L. Lafontant D.-E. Farris C. M. Ma Y. Urenia P. A. (2023). Assessment of heterogeneity among participants in the Parkinson’s progression markers initiative cohort using α-synuclein seed amplification: A cross-sectional study. Lancet Neurology 22 (5), 407–417. 10.1016/S1474-4422(23)00109-6 37059509 PMC10627170

[B41] Soto C. Saborio G. P. Anderes L. (2002). Cyclic amplification of protein misfolding: Application to prion-related disorders and beyond. Trends Neurosci. 25 (8), 390–394. 10.1016/S0166-2236(02)02195-1 12127750

[B42] Soto C. (2011). Prion hypothesis: The end of the controversy? Trends Biochem. Sci. 36 (3), 151–158. 10.1016/j.tibs.2010.11.001 21130657 PMC3056934

[B43] Srivastava A. Alam P. Caughey B. (2022). RT-QuIC and related assays for detecting and quantifying prion-like pathological seeds of α-synuclein. Biomolecules 12 (4), 576. 10.3390/biom12040576 35454165 PMC9030929

[B44] Stefani A. Iranzo A. Holzknecht E. Perra D. Bongianni M. Gaig C. (2021). Alpha-synuclein seeds in olfactory mucosa of patients with isolated REM sleep behaviour disorder. Brain 144 (4), 1118–1126. 10.1093/brain/awab005 33855335

[B45] The Ronald and Nancy Reagan Research Institute of the Alzheimer’s Association National Institute on Aging Working GroupAB (1998). Consensus report of the working group on: "Molecular and biochemical markers of alzheimer's disease". The Ronald and nancy reagan research Institute of the alzheimer's association and the national Institute on aging working group. Neurobiol. Aging 19 (2), 109–116. 10.1016/S0197-4580(98)00022-0 9558143

[B51] Toledo J. B. Abdelnour C. Weil R. S. Ferreira D. Rodriguez‐Porcel F. Pilotto A. (2022). Dementia with Lewy bodies: Impact of co‐pathologies and implications for clinical trial design. Alzheimer’s & Dement. 19 (1), 318–332. 10.1002/alz.12814 PMC9881193 36239924

[B46] Vanderstichele H. Bibl M. Engelborghs S. Le Bastard N. Lewczuk P. Molinuevo J. L. (2012). Standardization of preanalytical aspects of cerebrospinal fluid biomarker testing for Alzheimer’s disease diagnosis: A consensus paper from the Alzheimer’s biomarkers standardization initiative. Alzheimer’s Dementia 8 (1), 65–73. 10.1016/j.jalz.2011.07.004 22047631

[B48] Walker L. Stefanis L. Attems J. (2019). Clinical and neuropathological differences between Parkinson’s disease, Parkinson’s disease dementia and dementia with Lewy bodies – current issues and future directions. J. Neurochem. 150 (5), 467–474. 10.1111/jnc.14698 30892688

[B49] Wang Z. Becker K. Donadio V. Siedlak S. Yuan J. Rezaee M. (2021). Skin α-synuclein aggregation seeding activity as a novel biomarker for Parkinson disease. JAMA Neurol. 78 (1), 1–11. 10.1001/jamaneurol.2020.3311 32986090 PMC7522783

[B50] Wang Y. Hu J. Chen X. Wang S. Zhang C. Hu J. (2022). Real-time quaking-induced conversion assay is accurate for Lewy body diseases: A meta-analysis. Neurol. Sci. 43 (7), 4125–4132. 10.1007/s10072-022-06014-x 35312879

